# Repairing the Brain by SCF+G-CSF Treatment at 6 Months Postexperimental Stroke: Mechanistic Determination of the Causal Link Between Neurovascular Regeneration and Motor Functional Recovery

**DOI:** 10.1177/1759091416655010

**Published:** 2016-08-09

**Authors:** Lili Cui, Dandan Wang, Sandra McGillis, Michele Kyle, Li-Ru Zhao

**Affiliations:** 1 Department of Neurosurgery, State University of New York Upstate Medical University, Syracuse, NY, USA; 2 Department of Neurology, Louisiana State University Health Sciences Center, Shreveport, LA, USA

**Keywords:** brain repair, chronic stroke, G-CSF, hematopoietic growth factors, SCF, live brain imaging

## Abstract

Stroke, a leading cause of adult disability in the world, is a severe medical condition with limited treatment. Physical therapy, the only treatment available for stroke rehabilitation, appears to be effective within 6 months post-stroke. Here, we have mechanistically determined the efficacy of combined two hematopoietic growth factors, stem cell factor (SCF) and granulocyte-colony stimulating factor (G-CSF; SCF + G-CSF), in brain repair 6 months after cortical infarct induction in the transgenic mice carrying yellow fluorescent protein in Layer V pyramidal neurons (Thy1-YFP-H). Using a combination of live brain imaging, whole brain imaging, molecular manipulation, synaptic and vascular assessments, and motor function examination, we found that SCF + G-CSF promoted mushroom spine formation, enlarged postsynaptic membrane size, and increased postsynaptic density-95 accumulation and blood vessel density in the peri-infarct cavity cortex; and that SCF + G-CSF treatment improved motor functional recovery. The SCF + G-CSF-enhanced motor functional recovery was dependent on the synaptic and vascular regeneration in the peri-infarct cavity cortex. These data suggest that a stroke-damaged brain is repairable by SCF + G-CSF even 6 months after the lesion occurs. This study provides novel insights into the development of new restorative strategies for stroke recovery.

## Introduction

Stroke is a cerebrovascular disease in which brain tissue death (infarct) and neurological deficits occur from the sudden interruption of blood flow to a specific region of the brain. Stroke progresses through three phases: the acute, subacute, and chronic phase. The pathological profiles of the three phases appear to be quite different. Unlike in the acute and subacute phases, when massive neurons undergo primary and secondary damage (Parsons et al., 2000) in the chronic phase, a stroke patient’s neurological status becomes relatively stable and the surviving neurons establish new networks in an effort to take over the function of the dead neurons (Tombari et al., 2004; Carmichael, 2012; Cui et al., 2013; Zhao et al., 2013). The duration and pathological severity of the three phases vary between individuals and depend on the infarction size, infarct location, cerebrovascular collateral response, patient’s age, and medical comorbidities. Generally, the chronic phase begins 3 months after stroke onset (Hara et al., 1993; Parsons et al., 2000).

Stroke is an enormous public health problem and the leading cause of persistent disability worldwide. Today, there is a large population of chronic stroke patients in the world suffering from stroke-induced disability. A recent study shows that in 2010, there were 102 million disability-adjusted life-years lost in the world (Feigin et al., 2014). In the United States alone, about 6.6 million stroke survivors are suffering from persistent disability (Mozaffarian et al., 2015). Targeting brain repair in chronic stroke is a highly important but much less investigated field in stroke research. Speech and physical therapies appear to be the only therapies available for chronic stroke patients. Since it would be unfeasible for stroke patients to spend every hour with physical therapists for physical performance, developing alternatives, such as a pharmaceutical approach, to help in restoring motor function for stroke survivors is needed. Importantly, the therapeutic window for traditional physical therapy appears to be limited within 6 months after stroke onset (Hendricks et al., 2002; Schaechter, 2004). Over 50% of chronic stroke patients, who are discharged from rehabilitation therapy at 6 months post-stroke, still show significant motor impairment (Gresham et al., 1975; Hendricks et al., 2002; Kelly-Hayes et al., 2003). Currently, therapies that can further improve functional restoration 6 months after stroke occurs have not yet been developed.

Recently, we have demonstrated the therapeutic efficacy of stem cell factor (SCF) and granulocyte-colony stimulating factor (G-CSF) on brain repair and functional restoration in animal models of chronic stroke. SCF and G-CSF are well-characterized hematopoietic growth factors and play an essential role in controlling bone marrow stem cell growth, survival, and differentiation into blood cells (Welte et al., 1985; Zsebo et al., 1990). Increasing evidence, however, shows that SCF and G-CSF are also involved in neuronal plasticity, neuronal network formation, and neuronal function in learning and memory (Hirata et al., 1993; Motro et al., 1996; Katafuchi et al., 2000; Diederich et al., 2009; Su et al., 2013). Our earlier study revealed that systemic administration of combined SCF and G-CSF (SCF + G-CSF) 3.5 months after induction of cortical brain ischemia led to much greater functional improvement than SCF or G-CSF treatment alone (Zhao et al., 2007). However, it remains unanswered whether administration of SCF + G-CSF at a much-delayed time, 6 months after stroke, would be effective in brain repair.

Neurovascular network remodeling has been proposed to play an important role in stroke recovery (Moskowitz et al., 2010). Nuclear factor-κB (NF-κB), a transcription factor, is involved in synaptogenesis (Meffert et al., 2003; Memet, 2006; Boersma et al., 2011; Imielski et al., 2012) and angiogenesis (Stoltz et al., 1996). Our recent findings revealed that NF-κB mediates SCF + G-CSF-promoted neurite outgrowth in cultured primary cortical neurons (Su et al., 2013). The purpose of the present study was to determine whether administration of SCF + G-CSF at 6 months after experimental stroke would be effective in enhancing functional improvement and neurovascular network remodeling and whether NF-κB would be involved in the restorative process of SCF + G-CSF in such a delayed treatment.

## Materials and Methods

The procedures of animal experiments were approved by the Institutional Animal Care and Use Committee and performed in accordance with the National Institutes of Health Guide for the Care and Use of Laboratory Animals.

### Animals and Animal Model of Cerebral Cortical Ischemia

Four-month-old male Thy1-YFP-H transgenic mice (The Jackson Laboratory, Bar Harbor, ME) were subjected to cerebral cortical ischemia. In this transgenic line, only Layer V pyramidal neurons including the neuronal soma, axons, dendrites, and dendritic spines were labeled by YFP. Focal cerebral ischemia was induced by permanent occlusion of unilateral middle cerebral artery and common carotid artery (Piao et al., 2009; Cui et al., 2013). Briefly, mice were anesthetized by Avertin (i.p., 0.4 g/kg; Sigma-Aldrich, St. Louis, MO). After a midline incision in the neck, the right common carotid artery was exposed and ligated using a 6-0 silk suture. A craniotomy was made between the right eye and ear, and the right middle cerebral artery was cauterized. The rectal temperature was monitored and maintained at 37℃ throughout the surgery.

### Experimental Design and Drug Administration

**Figure 1.**
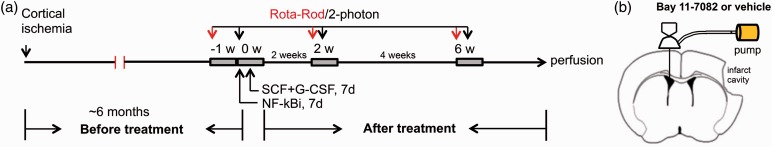
Experimental design and drug delivery. (a) Schematic diagram of experimental design. Focal brain ischemia was produced on 4-month old Thy1-YFP-H mice. SCF, G-CSF, and NF-κB inhibitor (Bay11-7082), were delivered about 6 months after stroke for 7 consecutive days. Rota-Rod test or 2-photon live brain imaging was performed before treatment, 2 and 6 weeks after treatment in two-paralleled experiments. The mice were perfused at the end of behavioral testing or live brain imaging. (b) Schematic diagram for lateral cerebroventricular delivery of Bay11-7082 through an osmotic minipump.

The schematic diagram of experimental design was shown in Figure 1(a). Approximately 6 months (6 ± 1 months) after cortical ischemia, mice were randomly assigned to one of the three groups: vehicle (stroke + vehicle), SCF + G-CSF (stroke + S + G), and SCF + G-CSF +NF-κB inhibitor (Bay11-7082; Sigma-Aldrich, St. Louis, MO; stroke + S + G + Bay). Recombinant mouse SCF (200 µg/kg/day; PeproTech, CA, USA), recombinant human G-CSF (50 µg/kg/day; Amgen, CA, USA), or an equal volume of vehicle solution was subcutaneously administered for 7 days. In S + G + Bay-treated mice, NF-κB inhibitor (Bay11-7082, 20 µM) was dissolved in 0.1% dimethyl sulfoxide and infused into the left lateral cerebroventricle (coordinates: 0.5 mm posterior to the bregma, 1.5 mm lateral to the midline) for 7 days through an Alzet® micro-osmotic pump (Durect Corporation, Cupertino, CA; Figure 1(b)). The 7-day delivery of NF-κB inhibitor was started 1 h before SCF + G-CSF treatment. As a vehicle control infusion, micro-osmotic pumps loaded with 0.1% dimethyl sulfoxide were implanted into the left ventricle of the mouse brain in other groups.

Age-matched Thy1-YFP-H mice without brain ischemia were used as intact control animals for imaging. Live brain imaging was repeatedly performed with a two-photon microscope before treatment (week 0), 2 and 6 weeks after treatment. Whole brain imaging was performed on the 4% paraformaldehyde-perfused brains at the end of the experiment. Motor function of additional stroke mice without live brain imaging was evaluated with a Rota-Rod before treatment as well as 2 and 6 weeks after treatment. Sample size was determined based on our experience in similar studies.

### Live Brain Imaging

Dendritic spines on the apical dendrites of Layer V pyramidal neurons in live mice (*n* = 3 in each group) were captured through a thinned-skull window above the peri-infarct cavity area using a two-photon microscope (Zeiss LSM 510; Zeiss, Deutschland, Germany). Thereafter, the thinned skull area was surrounded by skull fixture adhesive (Plastic one, Roanoke, VA) and filled with sterile saline. The two-photon microscope with an ultrafast Ti:sapphire laser was used to capture the apical dendrites. The YFP was excited at 920 nm wavelengths, and the z-stack images (∼30–40 µm, 1 µm interval) of three different fields were obtained through a 40× water-immersion objective (0.8 numerical aperture) and LSM 510 Image software. Two and six weeks after treatment, the live brain imaging was repeated at the same location of the same mice using the same method. The thinned skull window was carefully reprepared using the micro-surgical blade before imaging at 2 and 6 weeks after treatment. During the imaging process, the anesthesia was maintained by Avertin and body temperature was kept close to 37℃.

### Two-Photon Imaging on Perfused Brains (Whole Brain Imaging)

The apical dendrites and dendritic spines of Layer V pyramidal neurons on the contralesional and ipsilesional hemispheres were captured again on perfused brains. Briefly, at the end of live brain imaging (Week 6 posttreatment), the anesthetized mice were transcardially perfused with 0.1 M phosphate buffered saline (PBS; pH 7.4) followed by 4% paraformaldehyde in PBS. After perfusion, the brains were removed, postfixed in 4% paraformaldehyde overnight and cryoprotected in 30% sucrose at 4℃ until the brain samples sank to the bottom of vials. The brains were then immobilized by 1% agarose in a cap and imaged under the two-photon microscope in 0.1 M PBS using the same methods as in live brain imaging. Z-stack images were captured from three different fields surrounding the infarct cavity and the homotopic cortex in the contralesional hemisphere.

### Rota-Rod Test

A total of 25 stroke mice without brain imaging (*n* = 7 in vehicle group; *n* = 9 in S + G group; *n* = 9 in S + G + Bay group) were used for evaluation of motor function in a Rota-Rod test. Animals were placed on an accelerating Rota-Rod, and the time that mice remained on the rotating Rota-Rod was recorded. The rotation speed was slowly increased from 4 r/min to 40 r/min within 300 s. Before treatment, mice were trained on the Rota-Rod once a day for five consecutive days. After treatment, mice were tested three times a day on the Rota-Rod for 5 days. The average time riding on the Rota-Rod per each day was calculated and used for statistical analysis.

### Immunohistochemistry

Brains were cut into 30 -µm thick serial coronal sections through the entire brain with a cryostat. Brain sections across the infarct cavities were selected for immunohistochemistry. The brain sections were rinsed in 0.1 M PBS and incubated in a block solution containing 5% normal goat serum, 1% bovine serum albumin (IgG-free) (Jackson ImmunoResearch Labs, West Grove, PA), and 0.25% Triton X-100 in PBS for 1 h at room temperature to block the nonspecific staining. The sections were then incubated with primary antibodies in a mixture solution of 2.5% normal goat serum, 1% bovine serum albumin, and 0.25% Triton X-100 in PBS overnight at 4℃. Primary antibodies used in this study included rabbit anti-NF-kappa B p65 (1:200; Santa Cruz, Dallas, TX), mouse anti-neuronal nuclei (NeuN; 1:1000; EMD Millipore, Billerica, MA), mouse anti-postsynaptic density protein 95 (PSD-95; 1:250; Sigma-Aldrich, St. Louis, MO), and rat anti-cluster of differentiation 31 (CD31; 1:250; BD Biosciences, Franklin Lakes, NJ). After rinsing in PBS, the brain sections were incubated with fluorescence-conjugated secondary antibodies including DyLight 594-conjugated goat anti-rabbit, DyLight 488-labled goat anti-mouse, DyLight 649-conjugated goat anti-mouse IgG, and DyLight 633-conjugated goat anti-rat IgG (1:500; Jackson ImmunoResearch Labs, West Grove, PA) for 2 h at room temperature in dark. Brain sections were then mounted with a ProLong Gold Antifade reagent (Life technologies, Grand Island, NY) and imaged using a Zeiss confocal microscope (Zeiss LSM 510; Zeiss, Deutschland, Germany). Z-stack images with 1 -µm intervals for PSD-95 and 2 -µm intervals for CD31 were obtained through a 40× water-immersion objective (0.8 numerical aperture). Z-stack images were projected, and the reactive area of PSD-95 and CD31 were analyzed using ImageJ software.

### Image Analysis

Three or four randomly selected dendrites on each Z-stack image (3 Z-stack images acquired from three sites per brain, three to four dendritic segments per field, 20–30µm for each segment) were analyzed with the LSM 510 software. The widest spine head size on Z-stack slices was measured using the LSM 510 software. In this study, dendritic spines were classified into three types: the mushroom type (M-type, with well-defined necks and larger heads), the thin type (T-type, with thinner and longer necks and smaller heads), and uncertain type (U-type, without well-defined spine heads; Grutzendler et al., 2002; Neigh et al., 2004; Cui et al., 2013). Spines having heads exceeding 0.8 µm (>0.8 µm) were considered the M-type spines, otherwise the T-type (< 0.8 µm). A value of zero was used for determining the spine head size of U-type spines because of lacking spine heads. The total number of the spines and the total number of the subtype spines per 10 µm of each segment of selected dendrites (2∼4 segments per field, 20–30 µm for each segment) were calculated as the spine density.

### Statistics

Data of more-than-two groups were examined by ANOVA followed by Bonferroni or Dunn correction. Analysis of two-group data was performed with a Student’s *t*-test. Statistical significance was set at *p* < .05. Data were presented as mean ± SEM.

## Results

### SCF+G-CSF Treatment at 6 Months Post-Stroke Improves Motor Functional Outcome Through the Regulation of NF-κB

**Figure 2.**
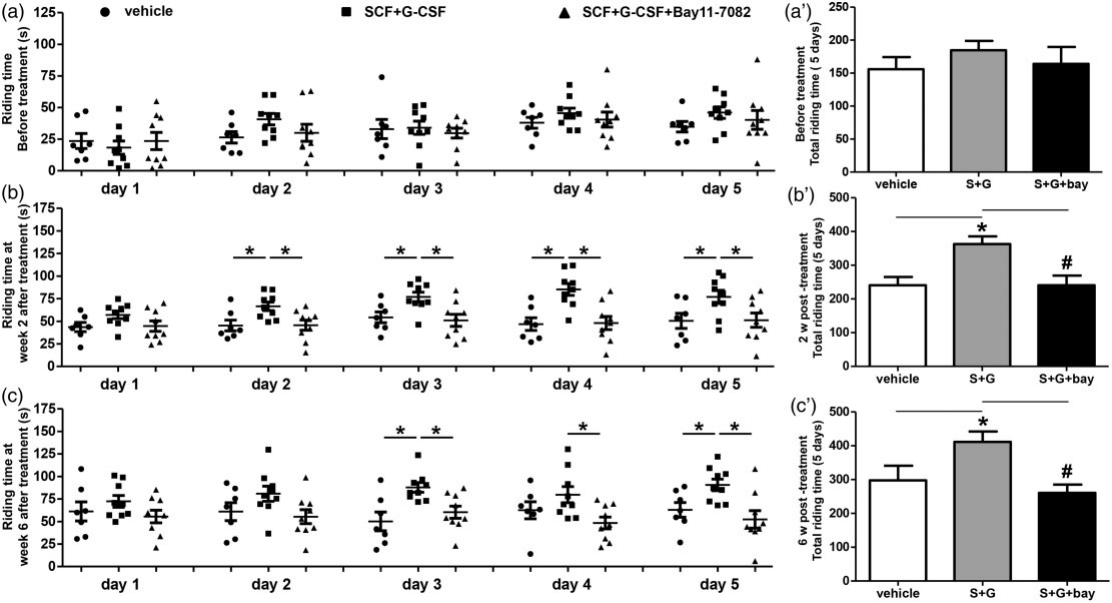
Delayed treatment of SCF + G-CSF in chronic stroke improves motor function via NF-κB. Motor function was evaluated by a Rota-Rod testing. Note that there are no differences in motor function among the 3 stroke groups before treatment (a) and (a'). SCF + G-CSF-improved motor function at 2 (b) and (b') and 6 (c) and (c') weeks after treatment is eliminated by NF-κB inhibitor (Bay11-7082). Mean ± SE. *p < .05, # *p* < .05.

In the pretest that was performed before treatment, all stroke mice remained on the rotating rod only for a short time; there were no differences in the performance of this pretest among the three experimental stroke groups (Figure 2(a) and 2(a’)).

Two weeks after treatment, however, SCF + G-CSF-treated mice showed a significant improvement in motor function as these mice remained on the rotating rod much longer than the vehicle control mice at Day 2, 3, 4, and 5 (*p* < .05; Figure 2(b) and 2(b’)). The mice that received treatment of SCF + G-CSF + NF-κB inhibitor did not show any improvement, as their ability to remain on the rotating rod was the same as the vehicle control mice through the entire testing process for 5 days. Similar results were observed 6 weeks after treatment (Figure 2(c) and 2(c’)). These data suggest that SCF + G-CSF treatment at 6 months post-stroke improves motor function depending on NF-κB.

### NF-κB Inhibitor Blocks SCF+G-CSF-Induced Activation of NF-κB in Cortical Neurons

To examine whether infusion of NF-κB inhibitor (Bay11-7082, 20 µM) in the left lateral ventricle of the brain could block SCF + G-CSF-promoted NF-κB activation in the cortical neurons in the right hemisphere, NF-κB inhibitor was continuously infused into the lateral ventricle of the left hemisphere for 5 days via an Alzet® micro-osmotic pump. One hour after delivering the NF-κB inhibitor, SCF + G-CSF was subcutaneously injected for 5 days. Mice that received treatment of vehicle solution, SCF + G-CSF, or SCF + G-CSF + NF-κB inhibitor (n = 3) were sacrificed through transcardial perfusion of 4% paraformaldehyde one day after the final injection of SCF + G-CSF.

**Figure 3.**
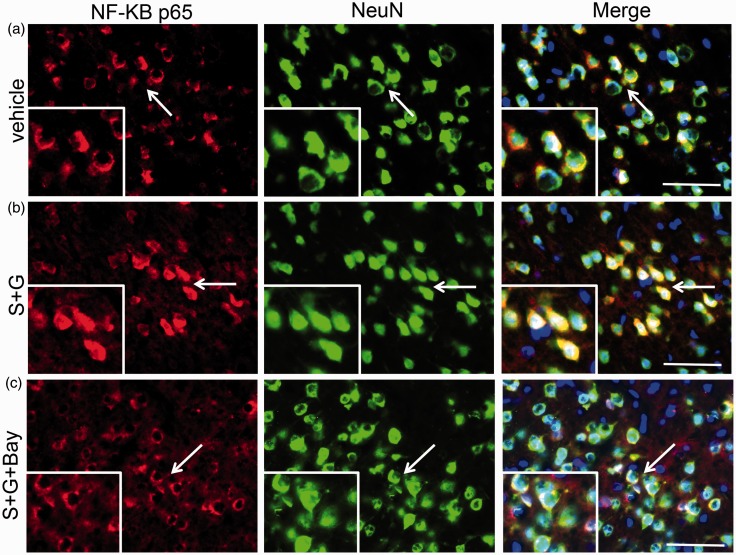
SCF + G-CSF treatment-induced NF-κB activation in cortical neurons is eliminated by lateral cerebroventricular infusion of NF-κB inhibitor. NF-κB inhibitor (Bay11-7082, 20 µM) was infused into the left lateral ventricle of the brain before and during SCF + G-CSF treatment. Confocal images show double immunofluorescent staining for NF-κB (red) and neurons (NeuN positive, green) in cortical neurons in the right hemisphere. Blue: DAPI, nuclear counterstaining. Note that the location of NF-κB in the neurons is changed by the interventions of SCF + G-CSF and NF-κB inhibitor. In vehicle controls (a), most NF-κB is located in neuronal cytoplasm (non-activation of NF-κB), whereas SCF + G-CSF treatment causes translocation of NF-κB from the cytoplasm to the neuronal nuclei (NF-κB activation). (b) Pre-treatment with NF-κB inhibitor, SCF + G-CSF-induced translocation of NF-κB into the nuclei is clocked. (c) Scale bar, 200 µm. Arrows indicate the location of the enlarged images in the box of each panel.

Through double immunofluorescent staining and confocal imaging, we found that the location of NF-κB in the cortical neurons of the right hemisphere was changed by the interventions of SCF + G-CSF and NF-κB inhibitor. In vehicle controls (Figure 3(a)), most NF-κB was located in neuronal cytoplasm (inactivated NF-κB), whereas SCF + G-CSF treatment caused translocation of NF-κB from the cytoplasm to the neuronal nuclei (activated NF-κB; Figure 3(b)). Pretreatment with NF-κB inhibitor, SCF + G-CSF-induced translocation of NF-κB into the nuclei were clocked (Figure 3(c)). This observation suggests that (a) systemic administration of SCF + G-CSF can promote NF-κB activation in cortical neurons and (b) infusion of NF-κB inhibitor into the *left* cerebroventricle is effective to eliminate the SCF + G-CSF-induced NF-κB activation in cortical neurons in the *right* hemisphere.

### SCF+G-CSF Treatment at 6 Months Post-Stroke Increases Mushroom Spine Formation in the Peri-Infarct Cavity Cortex Through NF-κB in Live Brain Imaging

It has been shown that motor activity in a Rota-Rod modifies dendritic spine formation (Yang et al., 2009). To prevent altering dendritic spines by repeated motor function tests with a Rota-Rod, the chronic stroke mice without behavioral tests were used for live brain imaging.

**Figure 4.**
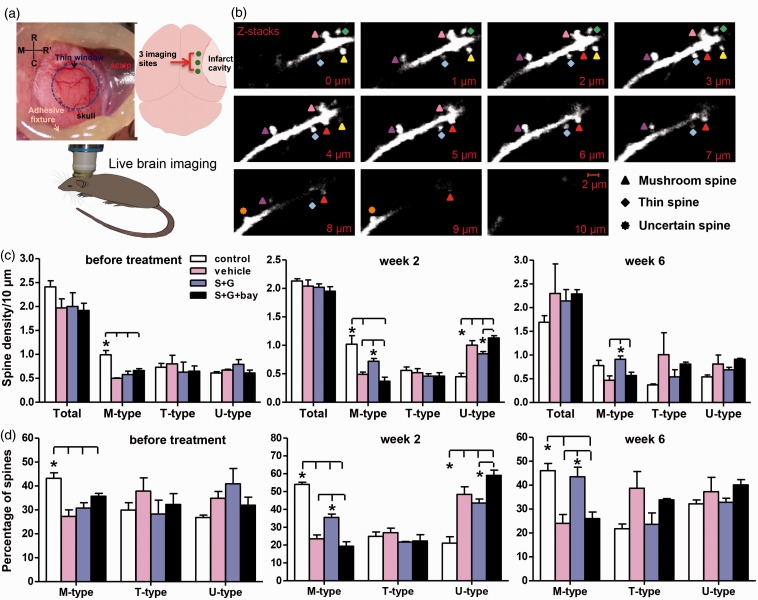
Delayed treatment of SCF + G-CSF in chronic stroke increases mushroom spine formation in the ipsilesional cortex through NF-κB in live brain imaging. (a) A thinned-skull window over the cortex outside the infarct cavity for 2-photon live brain imaging. R: rostral; C: caudal; M: midline; R’: right skull. (b) Representative z-stack images showing the subtypes of apical dendritic spines in Layer V pyramidal neurons. The widest spine head size for each spine was measured on the z-stack slices. (c) The spine density before treatment, 2 and 6 weeks after treatment. (d) The percentage of dendritic spines before treatment, 2 and 6 weeks after treatment. Note that the SCF + G-CSF-increased mushroom spine density (c) or the percentage of mushroom spines (d) in the cortex next to the infarct cavities is abolished by NF-κB inhibitor (Bay11-7082). Mean ± SE. *p < .05.

Convincing evidence has shown that neural network reorganization in the peri-infarct cortex is tightly related to functional improvement in the chronic phase of stroke (Tombari et al., 2004; Wang et al., 2010; Carmichael, 2012; Sharma and Cohen, 2012; Cui et al., 2015). To assess the dynamic changes of the dendritic spines on the apical dendrites of the Layer V pyramidal neurons in the peri-infarct cavity cortex, live brain imaging was repeatedly performed in the same mice before treatment (Week 0) as well as 2 and 6 weeks after treatment using a two-photon microscope through a thinned-skull window (Figure 4(a)). In Figure 4(b), the subtype of dendritic spines was illustrated through Z-stack images.

The total apical spine density in the peri-infarct cortex did not show differences between the intact controls and the three-stroke groups before or after treatment (Figure 4(c)). However, the M-type spines were dramatically changed by the stroke insult as well as by the treatment (Figure 4(c) and 4(d)). Before treatment, the M-type spines were significantly reduced in all the stroke groups as compared with the intact controls; but no difference was found among the three stroke groups. These data suggest that the reduction of M-type spines in the dendrites of Layer V pyramidal neurons surrounding the infarct cavity is related to cortical infarcts. The M-type spine reduction reflects synapse elimination or loss in the surviving neurons of peri-infarct cavity because of losing synaptic connections with the neurons that died in the infarct area.

Two weeks after treatment, however, the M-type spines were significantly increased by SCF + G-CSF as compared with the stroke-vehicle control groups (*p* < .05; Figure 4(c) and (d)). NF-κB inhibitor completely blocked the SCF + G-CSF-increased M-type spines (*p* < .05; Figure 4(c) and 4(d)). Six weeks after treatment, the SCF + G-CSF-increased M-type spines remained at a significantly elevated level (*p* < .05), which was similar to that of intact controls (Figure 4(c) and 4(d)). The effectiveness of SCF + G-CSF on enhancing M-type spine formation was fully prevented by the NF-κB inhibitor (*p* < .05; Figure 4(c) and 4(d)), suggesting a crucial role of NF-κB in mediating the SCF + G-CSF-induced regeneration of M-type spines.

It is worth noting that only SCF + G-CSF treatment caused a dynamic increase in M-type spines, whereas the M-type spines in the brains of stroke-vehicle controls showed no changes before treatment and after treatment. This observation indicates that the SCF + G-CSF-increased M-type spines are treatment-induced, but it is not influenced by the imaging manipulations.

U-type spines showed a transient alteration at Week 2 posttreatment. The U-type spines of all stroke groups appeared to be increased, while SCF + G-CSF treatment led to a trend toward decreasing the U-type spines 2 weeks after treatment. The SCF + G-CSF-decreased U-type spines were inhibited by the NF-κB inhibitor (*p* < .05; Figure 4(c) and 4(d)). U-type spines including stubby spines are considered to be the non-synaptic protrusions that represent either the newborn spines or degenerating synapses (Neigh et al., 2004). The precise mechanism underlying the transient changes in U-type spines of all stroke groups at 2 weeks posttreatment remains to be determined. It is unlikely that U-type spine formation is related to the manipulation of live brain imaging because the U-type spines in the intact brain remain unchanging as compared with the first imaging at Week 0 (Figure 4(c) and 4(d)). In fact, decreasing U-type spine formation and increasing M-type spine regeneration occurred simultaneously in the brains of SCF + G-CSF-treated chronic stroke mice at 2 weeks after treatment, suggesting a beneficial effect of SCF + G-CSF intervention in promoting U-type spine maturation to M-type spines.

T-type spines did not show any differences and changes among the experimental groups before or after treatment.

### SCF+G-CSF Treatment at 6 Months Post-Stroke Increases Mushroom Spine Density and Spine Head Size in the Peri-Infarct Cavity Cortex Through NF-κB in Whole Brain Imaging

**Figure 5.**
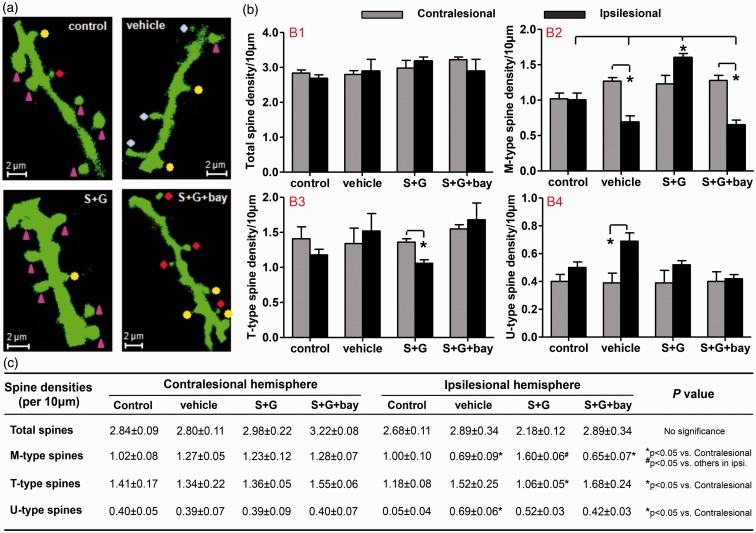
Delayed treatment of SCF + G-CSF in chronic stroke increases mushroom spine density in the peri-infarct cavity cortex through NF-κB 6 weeks after treatment in whole brain imaging. (a) Representative images of apical dendritic spines in Layer V pyramidal neurons in formalin-perfused brains. ▴ mushroom spines, ♦ thin spines, *uncertain spines. (b) Bar graphs of quantified dendritic spines in both hemispheres. Mean ± SE. (c) The table of quantified dendritic spines in both hemispheres. Mean ± SE. Control: intact mice, vehicle: vehicle-treated stroke mice, S + G: SCF + G-CSF-treated stroke mice, S+G+bay: SCF + G-CSF-treated stroke mice with NF-κB inhibitor (Bay11-7082) infusion to the brain.

To eliminate the potential influences caused by heart beating and breathing during the process of live brain imaging, the apical dendrites of Layer V pyramidal neurons in both hemispheres were scanned again with a two-photon microscope in the same animals that were imaged in live and perfused at the end of live brain imaging. The spine morphology and spine subtypes were identified through Z-stack images following the same criteria of live brain imaging (Figure 5(a)).

To validate the results of live brain imaging, spine density and spine type were determined in the cortex surrounding the infarct cavities. Consistent with the results of live brain imaging at 6 weeks after treatment, total spine density, and the densities of thin or uncertain spines in the peri-infarct cavity cortex did not show any differences among the intact control group and all stroke groups at 6 weeks after treatment (Figure 5(b)-1, -3, and -4; and 5(c)). The M-type spine density in the peri-infarct cavity cortex, however, showed quite a difference among all the experimental groups. SCF + G-CSF-treated mice displayed a significant increase in the M-type spines in the peri-infarct cavity cortex as compared with the vehicle control stroke mice and to the intact control mice in the corresponding cortex (*p* < .05; Figure 5(b)-2 and 5(c)). The SCF + G-CSF-increased M-type spines were totally blocked by NF-κB inhibitor (*p* < .05; Figure 5(b)-2 and 5(c)). These findings are similar to what we have observed in the live brain imaging in the cortex outside the infarct cavities. This observation further validates that SCF + G-CSF promotes regeneration of M-type spines in the Layer V pyramidal neurons adjacent to the infarct cavity through the regulation of NF-κB.

When comparing the dendritic spines of the Layer V pyramidal neurons in both hemispheres, we found that the total spine densities of both the peri-infarct cavity cortex and the contralesional cortex were not significantly different in each of the experimental groups (both the intact and chronic stroke groups; Figure 5(b)-1 and (c)), suggesting that neither chronic stroke nor SCF + G-CSF treatment changed the total spine densities in both hemispheres.

However, the spine types between the two hemispheres were changed by both the chronic stroke and SCF + G-CSF treatment. In the intact brain, M-type spines, T-type spines, and U-type spines were not different between the two hemispheres (Figure 5(b)-2∼4 and (c)). In the vehicle-control stroke mice, the M-type spines were significantly decreased, and the U-type spines were significantly increased in the peri-infarct cavity cortex in comparison with the contralesional cortex (*p* < .05; Figure 5(b)-2 and 5(b)-4 and 5(c)), indicating loss of synaptic connections in Layer V pyramidal neurons adjacent to the infarct cavities of chronic stroke brain. This synaptic loss may be due to the presynaptic neuron death in the infarct cortex during the acute phase of stroke. In the SCF + G-CSF-treated mice, the M-type spines showed a trend toward increasing, and the T-type spines were significantly reduced in the peri-infarct cavity cortex as compared with the contralesional cortex (*p* < .05; Figure 5(b)-2 and 5(b)-3, and 5(c)). Numerous studies have demonstrated that M-type spines are stable and functionally active spines, while T-type spines are not stable and are functionally silent spines (Matsuzaki et al., 2001; Kasai et al., 2010; Bosch and Hayashi, 2012). The SCF + G-CSF-induced simultaneous increases of M-type spines and decreases of T-type spines in the Layer V pyramidal neurons outside the infarct cavity suggest that SCF + G-CSF may promote T-type spine growing into the M-type spines. In the SCF + G-CSF + NF-κB inhibitor-treated mice, we found the same results as seen in the vehicle control stroke mice that the M-type spines were significantly less in the peri-infarct cavity cortex than in the contralesional cortex (*p* < .05; Figure 5(b)-2 and (c)), and that the T-type spines remained no different between the two hemispheres (Figure 5(b)-3 and 5(c)), suggesting that NF-κB inhibitor eliminates the SCF + G-CSF-induced modification of M-type and T-type spines in the peri-infarct cavity cortex.

**Figure 6.**
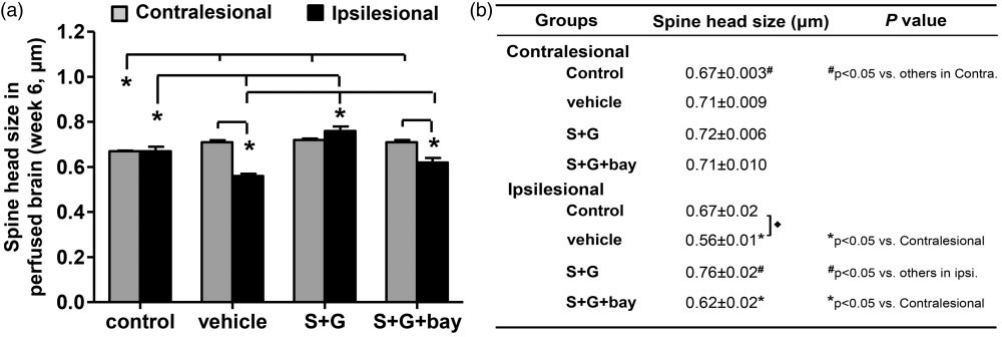
Delayed treatment of SCF + G-CSF in chronic stroke increases spine head size in the peri-infarct cavity cortex via NF-κB 6 weeks after treatment in whole brain imaging. (a) The bar graph of quantified dendritic head size in both hemispheres. (b) The table of quantified dendritic head size in both hemispheres. Spine head size: the widest dimension of the spine head. Mean ± SE. Control: intact mice, vehicle: vehicle-treated stroke mice, S + G: SCF + G-CSF-treated stroke mice, S+ G + bay: SCF + G-CSF-treated stroke mice with NF-κB inhibitor (Bay11-7082) infusion to the brain.

To further validate the efficacy of SCF + G-CSF in enhancing M-type spine regeneration, we measured the spine head size (the widest dimension of the spine head) in both hemispheres. In the ipsilesional cortex adjacent to the infarct cavities, the spine head size of the vehicle stroke controls was significantly reduced as compared with the corresponding cortex of intact brains (*p* < .05; Figure 5(a) and 5(b)), suggesting a cortical infarct-related reduction of spine head sizes in the chronic stroke brain. In addition, SCF + G-CSF significantly increased the spine head size in the peri-infarct cavity cortex as compared with the vehicle stroke controls and the SCF + G-CSF + NF-κB inhibitor-treated mice as well as to the intact controls (*p* < .05; Figure 6(a) and 6(b)). The spine head size of the SCF + G-CSF + NF-κB inhibitor-treated mice was similar to the vehicle stroke controls. These findings suggest that SCF + G-CSF enlarges the spine head size of Layer V pyramidal neurons in the peri-infarct cavity cortex in a lesion-, treatment-, and NF-κB-dependent manner.

When measuring the spine head size in the contralesional cortex, we found that the spine head size in the brains of all the chronic stroke mice were significantly larger than in the intact mouse brain (*p* < .05; Figure 6(a) and (b)), indicating that this increase is cortical infarct associated. This observation may suggest a lesion-induced reorganization of Layer V neuronal function in the contralesional cortex of chronic stroke brain. In addition, the spine head size in the peri-infarct cavity cortex of the vehicle controls and the SCF + G-CSF + NF-κB inhibitor-treated mice was significantly smaller than in the contralesional cortex (*p* < .05; Figure 6(a) and (b)). No difference in spine head size between the two hemispheres was observed in both the intact controls and the SCF + G-CSF-treated mice (Figure 6(a) and (b)), indicating reestablishment of the balance in the synaptic size between the two hemispheres by SCF + G-CSF. It has been demonstrated that postsynaptic membrane size is positively associated with synaptic transmission and function. (Matsuzaki et al., 2001; Kasai et al., 2010; Noguchi et al., 2011; Bosch and Hayashi, 2012) Therefore, these findings suggest that SCF + G-CSF may restore synaptic circuits in the surviving Layer V pyramidal neurons outside infarct cavities through the regulation of NF-κB.

### SCF+G-CSF Treatment at 6 Months Post-Stroke Increases PSD-95 Accumulation in The Peri-Infarct Cavity Cortex Through NF-κB

**Figure 7.**
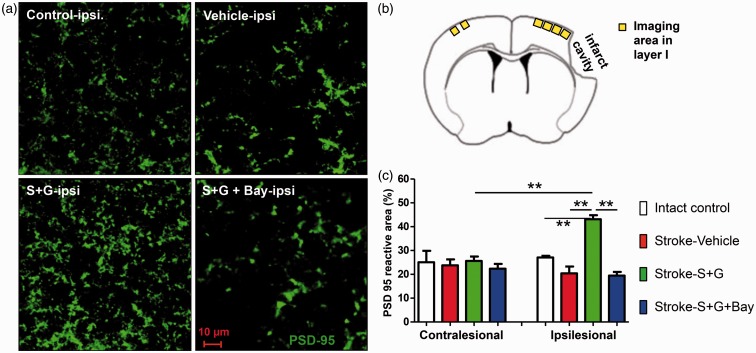
Delayed treatment of SCF + G-CSF in chronic stroke enhances PSD-95 accumulation in Layer I cortex outside the infarct cavities via NF-κB regulation. (a) Representative images for PSD-95 positive staining in Layer I cortex of the ipsilesional hemisphere. (b) Schematic diagram showing the imaging area in bilateral hemispheres. (c) Quantification of PSD-95 positive area in Layer I cortex. Control: intact control mice, vehicle: vehicle-treated stroke mice, S + G: SCF + G-CSF-treated stroke mice, S + G+bay: SCF + G-CSF-treated stroke mice with NF-κB inhibitor (Bay11-7082) infusion to the brain. Mean ± SE. **P < 0.01.

Next, we sought to validate the effectiveness of delayed SCF + G-CSF treatment on PSD-95 accumulation in the postsynapse of the chronic stroke brain. PSD-95, the postsynaptic element, was quantified in Layer I of the peri-infarct cavity cortex and the contralesional cortex homotopic to the peri-infarct cavity cortex (Figure 7). In the contralesional cortex, PSD-95 positive area was not different among all the experimental groups (intact controls and three stroke groups; Figure 7(c)), suggesting that SCF + G-CSF treatment has no effects in recruiting PSD-95 in the contralesional cortex. In the peri-infarct cavity cortex, however, SCF + G-CSF treatment resulted in significant increases in the PSD-95 positive area as compared with vehicle controls (*p* < .01; Figure 7(a) and 7(c)). The PSD-95 positive area of SCF + G-CSF + NF-κB inhibitor-treated mice showed no difference from the vehicle controls but exhibited a significant decrease as compared with the SCF + G-CSF-treated mice (*p* < .01; Figure 7(a) and (c)). These data indicate that NF-κB is required for the SCF + G-CSF-increased postsynaptic accumulation of PSD-95 in the peri-infarct cavity cortex.

We also noted that the PSD-95 positive area in the Layer I of the peri-infarct cavity cortex in SCF + G-CSF-treated mice were significantly greater than the contralesional cortex and the corresponding cortex of the intact controls, suggesting an overrecruitment of PSD-95 in the peri-infarct cortex of the SCF + G-CSF-treated mice (Figure 7(c)).

### SCF+G-CSF Treatment At 6 Months Post-Stroke Increases Angiogenesis in the Peri-Infarct Cavity Cortex Through NF-κB

**Figure 8.**
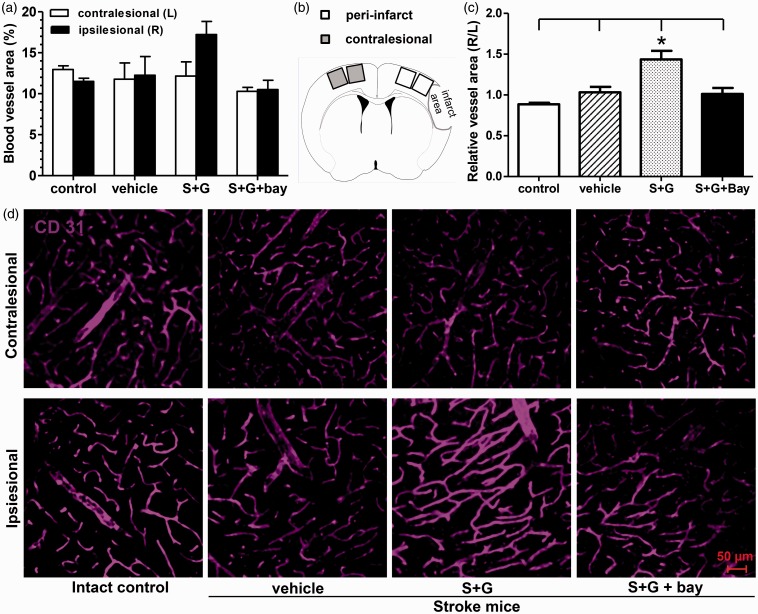
Delayed treatment of SCF + G-CSF in chronic stroke increases blood vessel density in the peri-infarct cortex through NF-κB. Blood vessels were detected by CD31 immunopositive staining. (a) Data of blood vessel density in both the ipsilesional and contralesional cortex. (b) Schematic diagram showing the imaging area in bilateral hemispheres. (c) Normalized data of blood vessel density. (d) Representative images for CD31 immunopositive staining in both the ipsilesional and contralesional cortex. Mean ± SE. *p < .05.

Emerging evidence shows that the structure and function of neurons and blood vessels are tightly coupled (Hamel, 2006; Lacoste and Gu, 2015). Therefore, in addition to demonstration of SCF + G-CSF-induced synaptic remodeling, we also determined the efficacy of SCF + G-CSF in angiogenesis when stroke mice were treated 6 months after induction of cortical infarcts. In the contralesional cortex, the blood vessel density did not show differences among the experimental groups (Figure 8(a)). In the peri-infarct cavity cortex, SCF + G-CSF treatment appeared to have an increase in blood vessel density. To prevent the potential influences by the processes of immunostaining and imaging, the ratio of ipsilesional (right hemisphere) to contralesional (left hemisphere) vascular density was calculated as a relative value of vascular density in the ipsilesional hemispheres (Figure 8(c)). Our data showed that the relative value of vascular density in the peri-infarct cavity cortex was significantly increased by SCF + G-CSF treatment when compared with the intact controls and the vehicle controls (*p* < .05; Figure 8(c)). Interestingly, inhibiting NF-κB before SCF + G-CSF treatment completely prevented the SCF + G-CSF-increased vascular density in the ipsilesional cortex, suggesting the involvement of NF-κB in regulating the SCF + G-CSF-enhanced angiogenesis in the chronic stroke brain.

To examine the direct effects of SCF + G-CSF treatment in activation of NF-κB in the cerebral endothelial cells, we utilized anti-NF-κB (p65) Maaser et al., 2001) and anti-CD31 antibodies to identify the location of NF-κB in the cerebral endothelial cells through immunofluorescence double staining in brain sections. Unlike the data showing obvious translocation of NF-κB into the nuclei of cortical neurons by SCF + G-CSF treatment (Figure 3), we did not observe the evidence that SCF + G-CSF treatment promoted NF-κB translocation from the cytoplasm to the nuclei (active form of NF-κB) of cerebral endothelial cells in the cortex (data not shown). This observation does not support that SCF + G-CSF treatment directly enhances cerebral angiogenesis through the regulation of NF-κB.

## Discussion

The findings of this study suggest that the time window of restorative therapy for chronic stroke is much longer than previously understood. As stated earlier, physical therapy is the only available treatment for stroke recovery. It has long been believed that the effective treatment window of physical therapy is within the first 6 months after stroke onset. Under standard rehabilitation care, motor recovery of stroke survivors appears to be the most rapid during the first month post-stroke, then it tends to slow down during the following months, and finally it plateaus by 6 months after stroke (Hendricks et al., 2002; Schaechter, 2004). After rehabilitation discharge at 6 months post-stroke, more than 50% of stroke survivors continue to exhibit motor impairment (Gresham et al., 1975; Hendricks et al., 2002; Kelly-Hayes et al., 2003). Recent pilot studies using advanced physical technologies have revealed the feasibility of motor functional improvement for chronic stroke patients 6 months or several years after stroke onset (Page et al., 2013; Small et al., 2013). In addition to the physical approach, our present study provides initial evidence showing that a pharmaceutical approach, SCF + G-CSF, promotes motor function and enhances brain repair when given 6 months after experimental stroke. Although it remains uncertain how the restorative intervention restores motor function in the very delayed chronic stroke phase, brain plasticity is most likely the mechanism behind the delayed recovery. Brain plasticity is an inherent capability of the brain to modify its function, structure, and microenviroment in response to stimuli and injuries. Convincing evidence shows that brain plasticity exists throughout an entire lifespan (Rosenzweig and Bennett, 1996; Johansson, 2000). Accordingly, the putative window of restorative therapy for stroke recovery could be extended to months and years after stroke onset.

The findings of this present study show that SCF + G-CSF treatment at 6 months postexperimental stroke leads to neurovascular remodeling in the peri-infarct cavity cortex, but not in the contralesional cortex. This observation is in line with clinical studies for stroke recovery in chronic stroke patients. Using noninvasive imaging techniques, numerous clinical studies have revealed that cortical reorganization activity in the ipsilesional somatosensorimotor cortex is positively correlated to motor functional recovery in chronic stroke patients (Tombari et al., 2004; Wang et al., 2010; Carmichael, 2012; Sharma and Cohen, 2012). Therefore, the therapies that enhance cortical network reorganization in the ipsilesional somatosensorimotor cortex could promote functional restoration in chronic stroke. A recent clinical study showed that transcranial direct current stimulation to increase excitability of the ipsilesional motor cortex improved motor functional outcome in chronic stroke patients (Lindenberg et al., 2010). Our present study provides new evidence that a pharmaceutical approach using SCF + G-CSF treatment in delayed stage of chronic stroke is able to improve motor function through increased neurovascular network rewiring in the peri-infarct cavity cortex.

Dendritic spines are the postsynaptic sites of the most excitatory synapses (Beaulieu and Colonnier, 1985; Nimchinsky et al., 2002). Growth and retraction of dendritic spines along with formation and elimination of synapses result in the rewiring of neural circuits (Bailey and Kandel, 1993; Knott et al., 2002; Trachtenberg et al., 2002; Holtmaat et al., 2005; Zuo et al., 2005; De Paola et al., 2006). Two-photon live brain imaging studies have revealed that the dendritic spines of Layer V pyramidal neurons in the somatosensory motor cortex are tightly engaged with neuronal function in an experience-, activity-, and injury-dependent manner. Trimming whiskers drove a novel sensory experience-induced stabilization of new spines in the barrel cortex (Holtmaat et al., 2006). New dendritic spine formation in the motor cortex was increased within 1 h after initiating a forelimb-reaching task (Xu et al., 2009) or within 2 days after motor learning through Rota-Rod training (Yang et al., 2009). Dendritic spine density in the peri-infarct cortex was reduced within 2 weeks and recovered 6 weeks after cortical brain ischemia (Brown et al., 2007). The findings of our present study reveal that SCF + G-CSF treatment 6 months after cortical infarcts does not affect total dendritic spine density but significantly increases mushroom spine formation in Layer V pyramidal neurons in the peri-infarct cavity cortex. Using combined approaches of two-photon uncaging of glutamate to the cortical pyramidal neurons and whole-cell recording in the live brains of adult mice, Noguchi et al. (2011) found that the mushroom spines were functionally active spines and were involved in forming functional neural circuits. In the present study, we have demonstrated that the SCF + G-CSF-increased mushroom spine regeneration on the apical dendrites of the Layer V pyramidal neurons in the peri-infarct cavity cortex is causally linked to motor function improvement, suggesting that SCF + G-CSF treatment at 6 months post-stroke may rebuild motor function-engaged neuronal circuits in the cortex adjacent to the infarct cavities.

It has been well documented that the spine size and synaptic function correlate positively; however, how spine size affects neuronal function are not fully understood (Bosch and Hayashi, 2012). The dendritic spines contain the postsynaptic density (PSD), which is an electron-dense disc-like structure (Bosch and Hayashi, 2012). PSD is composed of multiple proteins including neurotransmitter receptors, scaffolding proteins, ion channels, signal transduction molecules, and actin filaments (Sheng and Hoogenraad, 2007; Bosch and Hayashi, 2012). Enlarged spine head sizes house large PSD with higher numbers of α-amino-3-hydroxy-5-methyl-4-isoxazolepropionic acid (AMPA)-type glutamate receptors and large amount of PSD-95 and have long-term maintenance of synaptic strength (Steiner et al., 2008; Kasai et al., 2010; Bosch and Hayashi, 2012; Rochefort and Konnerth, 2012). Accordingly, the spines with large heads form functioning synapses (Kasai et al., 2010; Noguchi et al., 2011). PSD-95, a major scaffolding protein, is highly abundant in the PSD, regulates synaptic AMPA receptor trafficking and functioning (Beique et al., 2006; Bats et al., 2007) and governs spine genesis (El-Husseini et al., 2000; Ehrlich et al., 2007), suggesting a key role of PSD-95 in mediating synaptic formation, synaptic structure, and function. Dendritic spine expansion and maintenance is supported by actin polymerization coupled with PSD-95 increases in the spine head (Hu et al., 2011; Bosch and Hayashi, 2012). PSD-95 is required for activity-dependent spine growth. Knockdown of PSD-95 diminishes the AMPA receptor-mediated transmission and reduces spine growth (Ehrlich et al., 2007; Steiner et al., 2008; Xu et al., 2008). NF-κB is transcriptionally involved in regulation of synaptogenesis and functional circuit formation (Meffert et al., 2003; Memet, 2006; Boersma et al., 2011; Imielski et al., 2012). PSD-95 has been proven to be the transcriptional target of NF-κB to induce synaptogenesis. NF-κB enhances spine formation and increases spine head volume through PSD-95 (Boersma et al., 2011). Our present study shows that SCF + G-CSF treatment at 6 months postexperimental stroke augments mushroom spines, amplifies spine head size, and increases PSD-95 accumulation in the peri-infarct cavity cortex through NF-κB. Blockage of NF-κB eliminates the SCF + G-CSF-increased mushroom spine formation, spine head size enlargement, and PSD-95 density in the ipsilesional cortex, and also abolishes the SCF + G-CSF-improved motor function in chronic stroke. Thus, our data elucidate a key role of NF-κB in the SCF + G-CSF-induced synaptic remodeling in chronic stroke brain and demonstrate a dependent link between the SCF + G-CSF-promoted genesis and maintenance of large spines in Layer V pyramidal neurons outside the infarct cavities and the SCF + G-CSF-improved motor function in chronic stroke.

It is worth noting that SCF + G-CSF-treated animals show an “over-increase” of mushroom spines, spine head size, and PSD-95 density in the peri-infarct cavity cortex as these increases are much greater than the contralesional brain and the corresponding cortex of intact animals. This “over-increase” may reflect the SCF + G-CSF-induced robust enhancement of synaptic activity and synaptic strength in the surviving neurons outside the infarct cavity to take over the function of lost ones.

Reorganizing neurovascular unit has been proposed to play an important role in brain repair after stroke (Moskowitz et al., 2010). Besides rebuilding neural circuits via NF-κB, SCF + G-CSF treatment at 6 months postexperimental stroke also increases blood vessel density in the peri-infarct cavity cortex through NF-κB mediation. These data suggest that SCF + G-CSF-increased synaptic size and blood vessel density in the peri-infarct cavity cortex appear to be regulated via the same molecule, NF-κB. Other studies have revealed a close relationship between wiring neural circuits and building vascular networks. Dendritic spine remodeling in the peri-infarct cortex is tied to the neighboring new vasculature (Brown et al., 2007). Many molecules that promote neural wiring also enhance vascular generation; conversely, molecules that direct blood vessel construction also regulate neural circuit wiring (Carmeliet and Tessier-Lavigne, 2005). Interestingly, our present study shows that SCF + G-CSF also leads to an “over-increase” of blood vessel density in the peri-infarct cavity cortex when compared with the contralesional cortex and the corresponding cortex of intact animals. This finding couples the SCF + G-CSF-induced “over-increase” of mushroom spines, spine head size, and PSD-95 density in the peri-infarct cavity cortex, indicating that SCF + G-CSF simultaneously enhances both the synaptic circuit rewiring and vascular network generation in the peri-infarct cavity cortex through NF-κB. Blockage of the SCF + G-CSF induced synaptic and vascular remodeling in the peri-infarct cavity cortex by an NF-κB inhibitor the SCF + G-CSF-induced motor function improvement is eliminated. This study, therefore, provides evidence supporting the key contribution of neurovascular remodeling in brain repair 6 months after stroke.

Noninvasive imaging has revealed the unique feature in the brain, termed neurovascular coupling, which demonstrates that regional cerebral blood flow is regulated by local neural activity (Drake and Iadecola, 2007; Sandrone et al., 2014). Emerging evidence shows that cerebral vascular remodeling is dependent upon neural activity. Activating neurons release angiogenic factors that promote formation of new blood vessels (Lacoste and Gu, 2015). The findings of the present study also suggest that SCF + G-CSF-enhanced synaptic activity in the peri-infarct cavity cortex may lead to an increase in regional vascular generation. Although we observed that NF-κB inhibitor abolished both the SCF + G-CSF-increased mushroom spines, spine head size, and PSD-95 density in the peri-infarct cavity cortex and the SCF + G-CSF-increased blood vessel density in the same region, SCF + G-CSF-induced NF-κB activation was found in cortical neurons but not in the cerebral endothelial cells. These findings suggest that the SCF + G-CSF-enhanced synaptic rewiring in the peri-infarct cavity cortex via NF-κB could be the key player for driving angiogenesis in the same location.

In addition to neurons, pericytes and glial cells also release angiogenic factors that support regeneration of blood vessels (Lacoste and Gu, 2015). Whether SCF + G-CSF could regulate release of angiogenic factors from neurons, pericytes, and glial cells through NF-κB mediation to enhance angiogenesis would be open questions that need to be addressed in future studies. Previous studies have shown that SCF promotes angiogenesis in vitro via AKT and ERK (Matsui et al., 2004), and that G-CSF enhances neovascularization through neutrophil-released vascular endothelial growth factor (Ohki et al., 2005). It remains to be determined in future studies whether these putative pathways or regulations are involved in SCF + G-CSF-induced angiogenesis in chronic stroke.

In summary, using the combined approaches of live brain imaging, whole brain imaging, molecular manipulation, synaptic and vascular assessments, and motor function examination, this study provides initial evidence that a stroke-damaged brain at 6 months post-stroke could be repairable by pharmaceutical intervention—systemic administration of two hematopoietic growth factors, SCF + G-CSF. The SCF + G-CSF-induced neurovascular rewiring in the ipsilesional cortex is required for SCF + G-CSF-improved motor function in the delayed chronic phase. The unique approaches utilized in this study allow us to mechanistically define how SCF + G-CSF treatment repairs the brain in the delayed stage of chronic stroke and to clarify the tightly dependent link between SCF + G-CSF-promoted structural modifications of neurovascular networks in the peri-infarct cavity cortex and motor functional improvement in chronic stroke. This study provides evidence supporting the crucial role of neurovascular rewiring in the peri-infarct cavity cortex in stroke recovery in the chronic phase.
